# Ras-activated RSK1 phosphorylates EBP50 to regulate its nuclear localization and promote cell proliferation

**DOI:** 10.18632/oncotarget.7184

**Published:** 2016-02-03

**Authors:** HooiCheng Lim, Tzuu-Shuh Jou

**Affiliations:** ^1^ Graduate Institute of Molecular Medicine, National Taiwan University, Taipei, Taiwan; ^2^ Graduate Institute of Clinical Medicine, National Taiwan University, Taipei, Taiwan

**Keywords:** EBP50, nuclear localization, phosphorylation, proliferation, RSK1

## Abstract

Differential subcellular localization of EBP50 leads to its controversial role in cancer biology either as a tumor suppressor when it resides at the membrane periphery, or a tumor facilitator at the nucleus. However, the mechanism behind nuclear localization of EBP50 remains unclear. A RNA interference screening identified the downstream effector of the Ras-ERK cascade, RSK1, as the molecule unique for nuclear transport of EBP50. RSK1 binds to EBP50 and phosphorylates it at a conserved threonine residue at position 156 (T156) under the regulation of growth factor. Mutagenesis experiments confirmed the significance of T156 residue in nuclear localization of EBP50, cellular proliferation, and oncogenic transformation. Our study sheds light on a possible therapeutic strategy targeting at this aberrant nuclear expression of EBP50 without affecting the normal physiological function of EBP50 at other subcellular localization.

## INTRODUCTION

Cellular proteins exert distinct functions depending on their localizations in differential subcellular compartments, where they may possess preferential alterations in their post-translational modifications and/or interaction with other signaling molecules. By contrast, aberrant subcellular localization of proteins contributes to the development and progression of diseases, including metabolic diseases [1], neurodegenerative diseases [2], and cancer [3, 4]. An understanding of the mechanism for subcellular localization of disease-related signaling proteins is, thus, important for diagnosis and therapeutic interventions.

Ezrin-radixin-moesin (ERM)-binding phosphoprotein of 50 kDa (EBP50), a scaffold protein that is expressed at the apical surface of luminal organs, consists of two tandem postsynaptic density 95/disks large/zona occludens (PDZ) domains and a C-terminal ezrin binding site [5]. The PDZ domains of EBP50 preferably recognize the C-terminal PDZ binding motif (S/T)XL of its interacting partners to organize molecules into functional complexes at the membrane periphery [6]. As EBP50 was found to express mainly at the apical surface, early studies were heavily focused on its regulatory roles at the plasma membrane periphery, such as ion transporters activation [6], receptors recycling [7, 8], and microvillar assembly [9, 10]. EBP50 is localized at the apical brush border membrane of intestinal epithelial cells, in which it holds β-catenin at the cell-cell junction to maintain the integrity of epithelial tissues [11]. Depletion of EBP50 causes membrane displacement of phosphatase and tensin homolog (PTEN) and nuclear translocation of β-catenin, events contributing to polarity loss and increased proliferation in a three-dimensional model of developing human intestinal glands [12]. Furthermore, EBP50 interacts with pleckstrin-homology domain leucine-rich repeat protein phosphatases (PHLPP) and scaffolds it with PTEN to form a tumor suppressor complex, which inhibits the oncogenic phosphatidylinositol-3-OH kinase (PI3K)-Akt signaling pathway [13]. These findings had once lead to the conclusion that EBP50 is a tumor suppressor. Nevertheless, EBP50 was later found localized at the nucleus in cholangiocarcinoma line Mz-Ch-A1, colon adenocarcinoma SW480 cell lines, as well as specimens of hepatocellular, colorectal and breast cancer [14-19]. However, the mechanism governing aberrant nuclear localization of EBP50 is undefined. Re-distribution of EBP50 from apical membrane to cytoplasmic and nuclear localization is reported to be strongly associated with the invasive phenotype of colorectal cancer [15-17]. The result of these studies highlights the urge for exploring the mechanism underlying nuclear localization of EBP50 in various carcinomas.

The p90 ribosomal S6 kinase (RSK) is a family of AGC kinases consists of four isoforms (RSK1-4), which have two kinase domains, an extracellular signal-regulated kinase (ERK) docking motif (D domain), and a C-terminal PDZ binding motif [20, 21]. The C-terminal kinase domain (CTKD) of RSK is responsible for its activation through autophosphorylation, whereas the N-terminal kinase domain (NTKD) is essential for the phosphorylation of its substrates [20]. Upon stimulation of cells with growth factors, ERK is phosphorylated downstream of the Ras cascade. Then, activated ERK initiates activation of RSK, *via* docking at the D domain and phosphorylating RSK at threonine 573 [22, 23]. Activated RSK has both cytoplasmic and nuclear substrates, where it regulates cell proliferation and growth [24, 25], apoptosis [26], and cell migration [27].

Here, we report that RSK1, which functions downstream of the Ras-ERK signaling cascade, binds to, phosphorylates EBP50, and leads to its nuclear mislocalization. Specifically, RSK1 phosphorylates EBP50 at threonine 156 (T156) residue in a cell cycle-dependent manner, which is important for nuclear translocation of EBP50 to facilitate cellular proliferation and transformation.

## RESULTS

### Ras-RSK1 signaling promotes nuclear localization of EBP50

**Figure 1:**
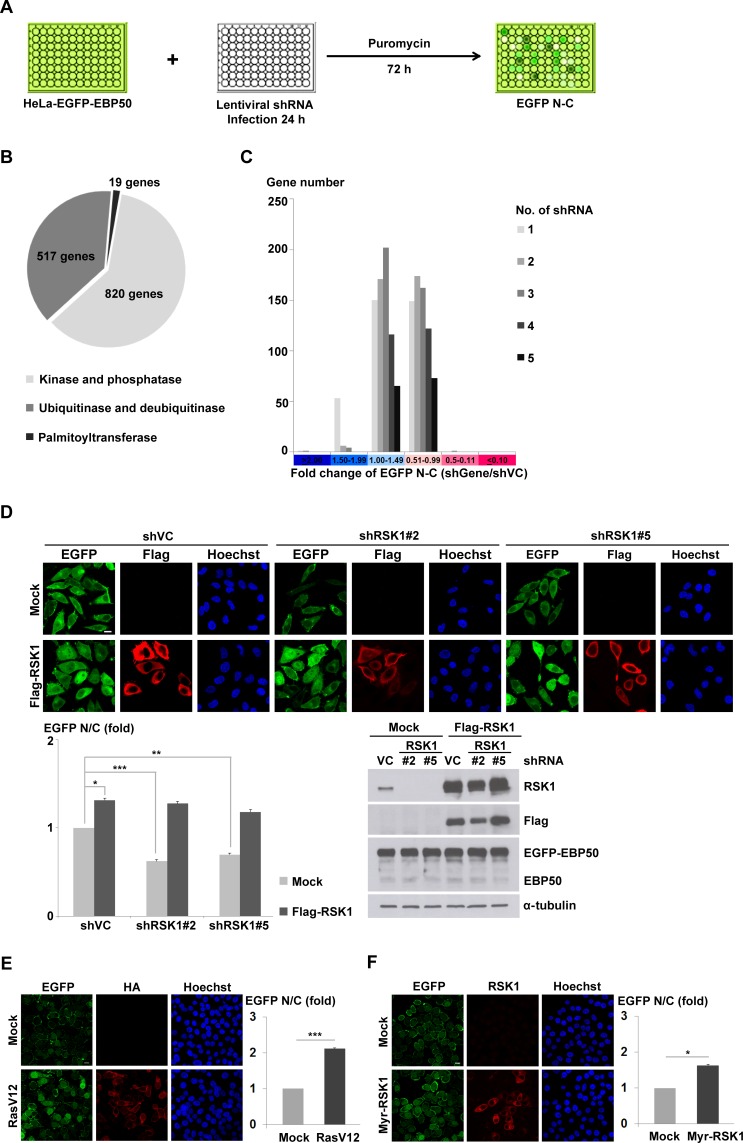
Ras-RSK1 signaling regulates nuclear localization of EBP50 **A.** Schematic diagram of the RNAi screening that was subsequently coupled with image analysis to search for genes involved in the nucleocytoplasmic transport of EBP50. **B.** Totally 1356 genes composed of three major enzymatic subclasses responsible for protein phosphorylation, ubiquitination, and palmitoylation were screened for their potential effects on the nucleocytoplasmic shuttling of EBP50. **C.** The nuclear and cytosolic (N-C) difference of EGFP signals after normalization with that from the mock-transduced cells (shVC) was presented according to the distribution of shRNA hits in the designated categories for each gene. **D.** Flag-tagged mouse RSK1 (Flag-RSK1) was introduced as the RNAi-resistant rescue construct to the HeLa-EGFP-EBP50 cells which were separately infected with two independent clones of lentiviruses expressing shRNAs against RSK1. Immunofluorescence images were analyzed for N/C ratio of EGFP signal (left lower panel). Western blotting analysis confirmed the knockdown efficiency of endogenous RSK1 and successful re-expression of Flag-RSK1 (right lower panel). α-tubulin was examined as a loading control. HeLa-EGFP-EBP50 cells were transfected with a plasmid encoding constitutive active Ras (HA-RasV12) **E.**, constitutive active RSK1 (Myr-RSK1) **F.**, or the empty vector (mock). The cells were then fixed at 24 h post-transfection, stained with anti-HA **E.** or anti-RSK1 antibody **F.**, and then images were captured and N/C ratio of EGFP signal were quantified. Scale bar: 10 μm. Data are means ± SEM of 90-200 cells. * *p* < 0.05, ** *p* < 0.01, *** *p* < 0.001, Student's *t*-test.

The reported interacting partners of EBP50 was reviewed and categorized based on their subcellular localization [28] (Figure S1A). In addition to the membrane and cytoplasmic proteins, EBP50 interacts with proteins that shuttle between cytoplasm and nucleus, including G protein-coupled receptor kinase 6A [29], epidermal growth factor receptor [30], and Yes-associated protein [31], as well as the nucleus-localized Wnt-responsive transcription factor TCF1 [15]. This finding is in accordance with the recent understanding about EBP50, a scaffold protein that not only organizes molecular complexes at the membrane periphery but is also found localized at the nucleus of cultured cells and carcinoma specimens [14, 15, 19]. To discover the signaling pathway governing the nucleocytoplasmic transport of EBP50, we generated a HeLa cell clone that stably expressed enhanced green fluorescent protein (EGFP)-tagged EBP50 (HeLa-EGFP-EBP50). This cell line was subjected to an arrayed RNA interference (RNAi) screening (Figure 1A) based on the premise that post-translational modification of EBP50, a highly phosphorylated protein, would likely affect its subcellular localization. Totally 1356 genes encoding enzymes that are involved in protein phosphorylation, ubiquitination and palmitoylation were included in this screening (Figure 1B). The changes in the distribution of EGFP signals between nucleus and cytoplasm of shRNA lentiviral-infected HeLa-EGFP-EBP50 cells were automatically quantified on a platform as described in the methodology section and scored for potential modulators in the nuclear trafficking of EBP50 (Table S1). As expected, most of the genes included in this kinome-focused analysis had an insignificant effect on the nucleocytoplasmic transport of EBP50 (Figure 1C). A total of 11 genes were identified as the potential regulator in nuclear export of EBP50 as knockdown of each gene resulted in a more than 50% increase of nuclear EBP50 (Figure S1C). Conversely, depletion of RSK1 by two independent lentiviral clones significantly reduced nuclear accumulation of EBP50, and this RNAi-induced effect could be rescued by introduction of cross-species and RNAi-resistant RSK1 cDNA (Figure 1D). RSK1 is the downstream effector of the Ras-ERK signaling pathway which phosphorylates many nuclear transcriptional factors [32, 33]. Accordingly, overexpression of the constitutively active oncogenic Ras (RasV12) promoted nuclear localization of EBP50 (Figure 1E); however, this effect of RasV12 was inhibited by a pharmacological inhibitor of RSK, BI-D1870 (Figure S1D). Furthermore, increased nuclear EBP50 was also observed in cells that overexpressed constitutive active mutant of RSK1 (Figure 1F). In summary, these results demonstrate that Ras-RSK1 signaling promotes nuclear localization of EBP50.

### RSK1 binds to EBP50 through a PDZ domain interaction

**Figure 2:**
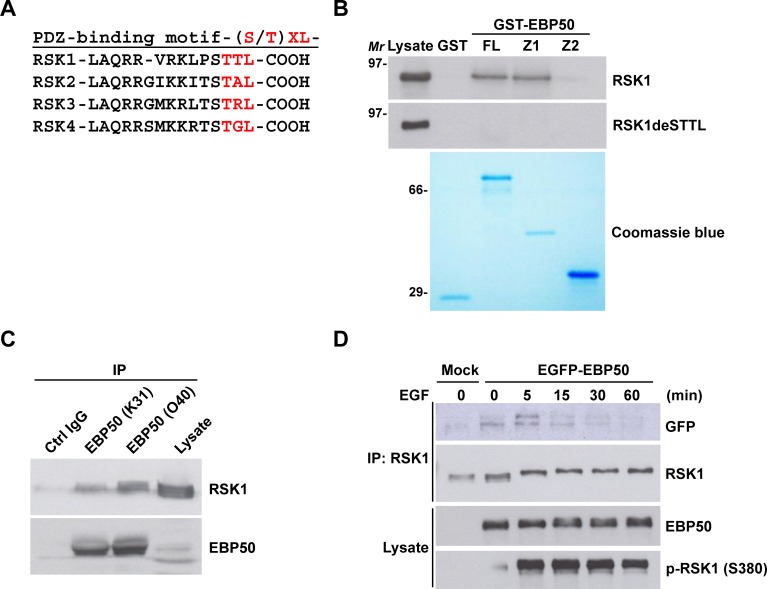
RSK1 binds to EBP50 through a PDZ binding motif and PDZ domain interaction which is subjective to signaling regulation **A.** The C-terminal sequences of all four RSK isoforms contain the putative PDZ binding motif, (S/T)XL, for the tandem repeated PDZ domains of EBP50. **B.** Cell lysates from HEK293 cells transiently expressing Myc-tagged full-length RSK1 (RSK1) or a PDZ binding motif deletion mutant (RSK1deSTTL) were subjected for *in vitro* pull-down assay with GST-fused full-length (FL), first PDZ domain (Z1) or second PDZ domain (Z2) EBP50 construct. The pulled down materials were separated by SDS-PAGE and analyzed by Western blotting using anti-Myc antibody. The coomassie blue-stained gel showed the expression and comparable loading of each recombinant protein used in the assay. Molecular marker (*Mr*): kDa. **C.** Endogenous EBP50 was immunoprecipitated (IP) from HeLa cells using two independent anti-EBP50 antibodies (K31 and O40), and co-immunoprecipitated RSK1 was detected by Western blotting using anti-RSK1 antibody. K31 and O40 were our in-house anti-EBP50 antibodies. They were generated by immunizing rabbits with a synthetic peptide (MARERAHQKRSSKRC for K31 and LQKLGVPVREELLRAQC for O40) and further purified using affinity chromatography. Immunoprecipitation using the antigenic peptide reabsorbed O40 immune serum was included as a negative control. **D.** EBP50-knocked down HeLa cells that stably re-expressed EGFP-EBP50 or empty vector were serum starved for 20 h, and stimulated with EGF (50 ng/ml) for the indicated intervals. Then, RSK1 was immunoprecipitated from the harvested lysates followed by Western blotting using anti-GFP antibody.

EBP50 preferably recognizes the PDZ binding motif (S/T)XL, through its PDZ domains, commonly found in a broad range of its interacting partners which is also noticed in RSKs (Figure 2A). To investigate the potential interaction between EBP50 and RSK1, GST-fused full-length EBP50 and its truncated mutants were employed to interact with RSK1 that was expressed transiently in HEK293 cells in a pull-down assay. Indeed, RSK1 binds to the first PDZ domain of EBP50 through its C-terminal PDZ binding motif as deletion of the terminal 4 amino acids from RSK1 abolished this interaction (Figure 2B). The physical association between endogenous proteins of RSK1 and EBP50 was confirmed by co-immunoprecipitation (Figure 2C). Additionally, the interaction of RSK1 with EBP50 was also examined through reciprocal co-immunoprecipitation studies in HeLa cells (Figure S2A-B). Interestingly, the association of RSK1 with EBP50 was apparently stimulated by epidermal growth factor (EGF) in HeLa cells which quickly fell to the resting status within 60 min (Figure 2D). In addition, RSK2 and RSK3 also bind to the first PDZ domain of EBP50 (Figure S2C). Collectively, RSK1 binds to EBP50 at its first PDZ domain in a dynamic and tightly regulated manner.

### RSK1 phosphorylates EBP50 at the RXRXXpS/T motif

**Figure 3:**
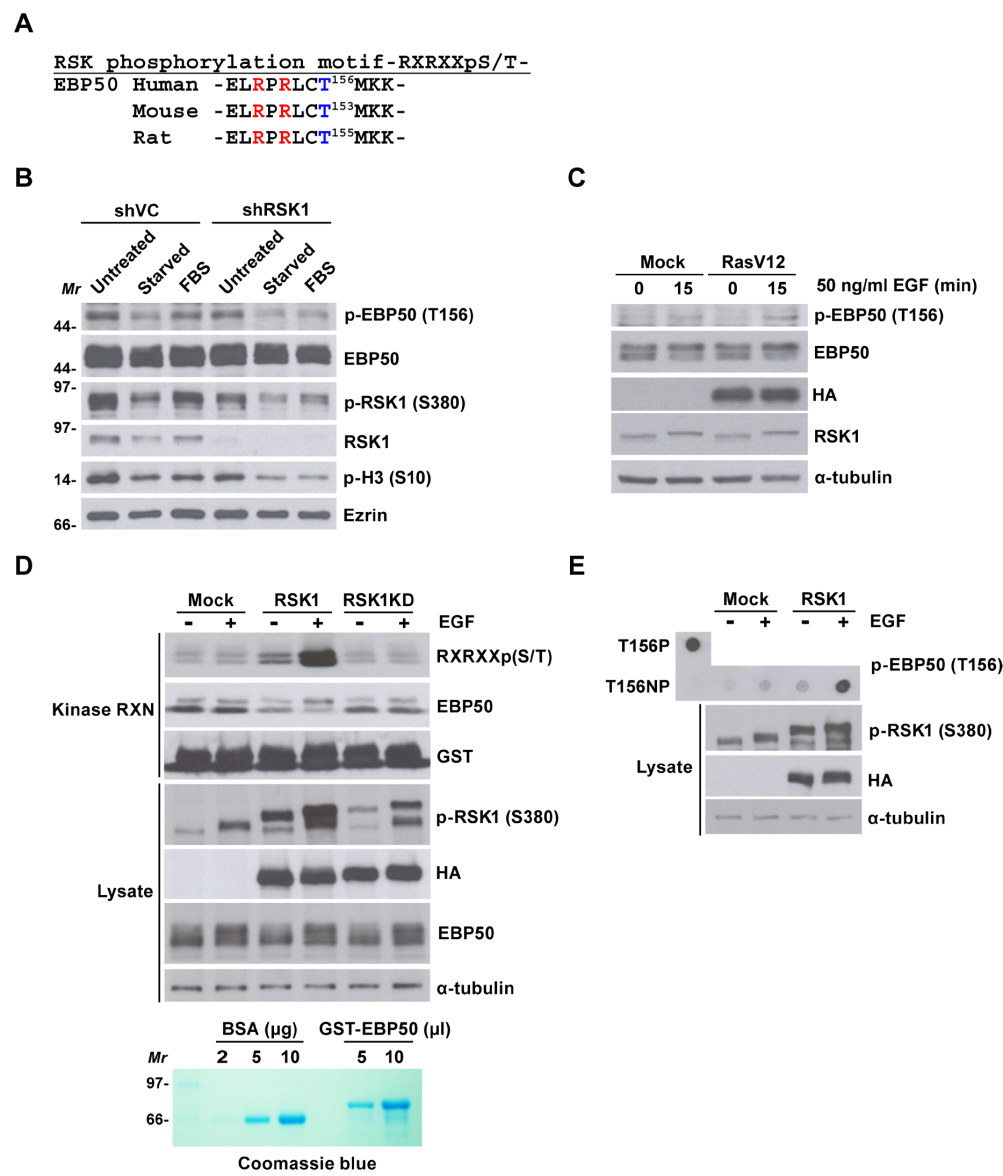
RSK1 phosphorylates EBP50 at T156 within a consensus RXRXXpS/T motif **A.** Mammalian EBP50 conserves a consensus phosphorylation motif of RSK1 substrates, RXRXXpS/T. **B.** RSK1-knocked down HeLa cells (shRSK1) or mock-transduced cells (shVC) were grown in complete media (untreated), serum-starved, or re-stimulated with 10% (v/v) serum for 7 h (FBS). EBP50 phosphorylation was analyzed by immunoblotting using phospho-T156 antibody. Knock down efficiency and activation of RSK1 was analyzed using anti-RSK1 antibody and phospho-S380 antibody, respectively. Phosphorylation of histone H3 at S10 served as the mitotic marker. Molecular marker (*Mr*): kDa. **C.** HEK293 cells were transfected with HA-RasV12 or empty vector, serum starved, and stimulated with EGF before EBP50 phosphorylation was analyzed. **D.** HEK293 cells were transfected with HA-tagged RSK1 (RSK1), its kinase dead mutant (RSK1KD), or an empty vector, serum starved, and stimulated with EGF. The immunoprecipitated RSK1 was incubated with 5 μl purified GST-EBP50, and phosphorylation of EBP50 was analyzed by immunoblotting using an antibody recognizing the RXRXXpS/T motif. Molecular marker (*Mr*): kDa. **E.** HEK293 cells were transfected with HA-RSK1 or empty vector, serum starved, and stimulated with EGF. The immunoprecipitated RSK1 was incubated with synthetic peptide of EBP50 (T156NP). The samples were then processed for dot blot analysis using phospho-T156 antibody. Synthetic phosphopeptide of EBP50 (T156P) was included as a positive control. Ezrin or α-tubulin was examined as a loading control.

Ras-ERK-dependent activation of RSK induces phosphorylation of proteins possessing the RXRXXpS/T consensus phosphorylation motif [34]. This phosphorylation motif is also present in human, rat and mouse sequences of EBP50 (Figure 3A). Conservation of the RXRXXpS/T motif among mammalian EBP50 signifies the potential of EBP50 as a candidate substrate of AGC kinases such as RSK1 and its implication in certain essential biological function. A previous proteomic approach exploiting an antibody recognizing RXRXXpS/T motif had identified EBP50 as a potential substrate phosphorylated at this motif (http://www.phosphosite.org/proteinAction.do?id=4000&showAllSites=true). To definitively examine if EBP50 could serve as a substrate of RSK1, phospho-T156 antibody that recognizes phosphorylation of human EBP50 at the RXRXXpS/T motif was generated. Dot blot analysis demonstrated that this antibody recognizes the antigenic phosphopeptide, but not the control unphosphorylated peptide (Figure S3A-B). EBP50 phosphorylation was then analyzed in HeLa cells that re-stimulated with serum after 48 h of starvation. The result shows that, following serum stimulation for 7 h, EBP50 was phosphorylated at T156 up to the level as when cells were grown in optimal media (Figure 3B). By contrast, serum-stimulated phosphorylation of EBP50 was significantly less in RSK1-knocked down cells (Figure 3B). Furthermore, overexpression of the oncogenic RasV12 mutant enhanced phosphorylation of EBP50 at T156 in HEK 293 cells (Figure 3C). These results indicate the essential role of Ras-RSK1 signaling for phosphorylation of EBP50 at T156. To demonstrate specifically that RSK1 indeed is the kinase directly responsible for phosphorylation of EBP50 at the RXRXXpS/T motif, RSK1 was immunoprecipitated from HEK293 for an *in vitro* kinase assay. This assay showed that EGF-activated wild type RSK1, but not the kinase dead mutant, could phosphorylate recombinant EBP50 at the RXRXXpS/T motif (Figure 3D). RSK1-mediated phosphorylation of EBP50 at T156 was further confirmed by an independent *in vitro* kinase assay, using synthetic peptide as substrate (Figure 3E). In summary, RSK1 phosphorylates EBP50 at T156 that lies within the conserved RXRXXpS/T motif. However, RSK2 and RSK3, bound to, but could not phosphorylate EBP50 (Figures S2C and S3C).

### Phosphorylation of EBP50 at T156 is related to increased nuclear localization

**Figure 4:**
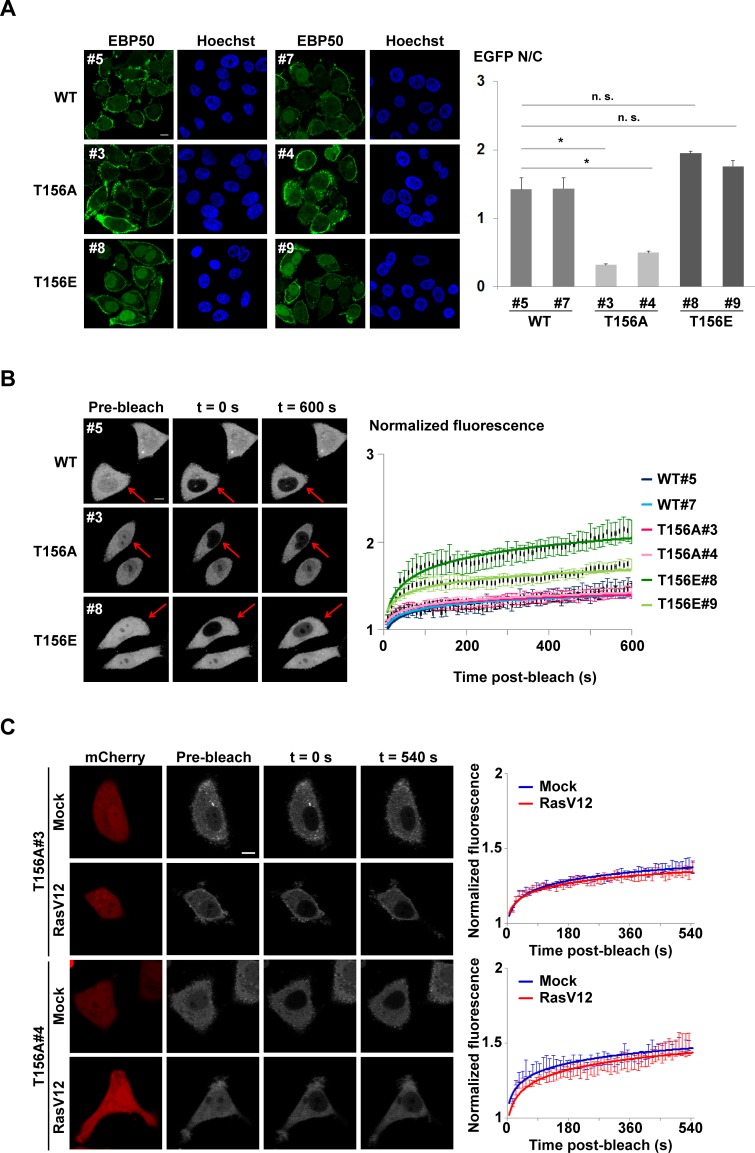
Phosphorylation of EBP50 at T156 is related to increased nuclear localization **A.** Immunofluorescence staining to analyze the subcellular distribution of EBP50 and its phospho-mutants in cells grown in complete media. Quantitative data are means ± SEM of 50-60 cells. * *p* < 0.05, n.s. not significant, Student's *t*-test. **B.** Photobleaching nuclear import assay on phospho-mutants of EBP50. Fluorescence images of typical cells from each clone at pre- or post-bleach times and at experimental end points. Quantitative data of the indicated photobleached-cells (red arrow) for recovery of fluorescence are means ± SEM of 14 cells for each clone. Scale bar: 10 μm. **C.** Photobleaching nuclear import assays on two independent HeLa cell clones stably expressing EGFP-tagged T156A EBP50 mutant that were transfected with HA-RasV12 or an empty vector. These cells were grown in complete medium with 10% FBS and treated with LMB (10 ng/ml) for an hour before experiments. RasV12-expressing cells were selected based on co-transfection with a plasmid that expressed mCherry. Fluorescence images of representative cells from each clone at pre-, post-bleach, and at end points were shown. Quantitative data of the photobleached-cells for recovery of nuclear fluorescence are means ± SEM of 8 cells for each time points. Scale bar: 10 μm.

To test the hypothesis that RSK1 regulates nuclear localization of EBP50 through phosphorylation, phospho-mutant constructs of EBP50 at T156 was generated (Figure S4A) and then stably expressed in endogenous EBP50-knocked down HeLa cells. Western analysis indicated the expression level of endogenous EBP50 was efficiently replaced by the phospho-mimetic (T156E) or phospho-resistant (T156A) mutants (Figure S4B). For cells grown in optimal media containing 10% (v/v) serum, the T156A mutant demonstrated a marked decrease in nuclear accumulation; while slightly higher population of T156E mutant was observed at the nucleus compared to wild type EBP50, as analyzed by quantitative immunofluorescence staining (Figure 4A), probably reflecting the possible involvement of other mechanisms which act on a T156 phosphorylation-independent way to govern EBP50 trafficking. The function of T156 on the dynamic of EBP50 subcellular distribution was also investigated *in vivo* using fluorescence recovery after photobleaching (FRAP) approach. To specifically study the nuclear import of EBP50, serum-starved cells at resting state were treated with leptomycin B (LMB), an inhibitor of proteins nuclear export [35], one hour prior to experiments. LMB treatment in HeLa cells leads to accumulation of EBP50 in the nucleus (Figure S4C). For FRAP experiments performed on serum-starved resting cells not favored for phosphorylation at T156, the nuclear import rate of T156A mutant was similar to that of wild type protein. However, the T156E mutant was recovered inside the photobleached-nuclear area much faster (Figure 4B). In addition to the clones shown in the left panel of Figure 4B, additional independent clones also displayed the same trend in term of their nuclear trafficking behaviors that indicated this result is not a phenomenon due to clonal bias (Figure S4D). Moreover, overexpression of RasV12 did not lead to any significant change on nuclear trafficking of two independent phospho-resistant T156A mutant clones grown in complete medium (Figure 4C); however, RSK1-knocked down diminished the fluorescent recovery of nuclear EBP50 in RasV12-expressing cells (Figure S4E), thus suggesting that RSK1-dependent phosphorylation at T156 is specific for nuclear localization of EBP50 downstream of Ras pathway.

### Cell cycle-dependent phosphorylation of EBP50 is crucial for growth of HeLa cells

**Figure 5:**
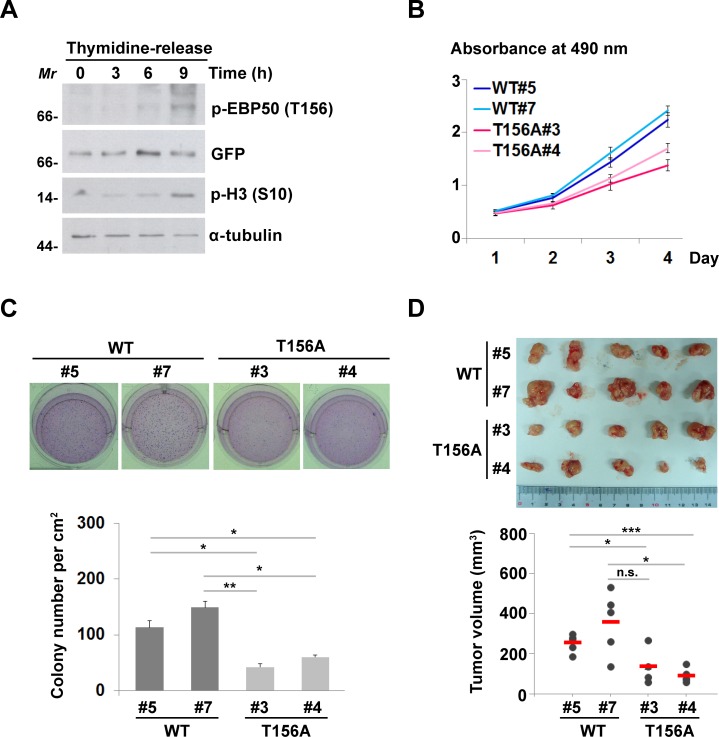
Cell cycle-dependent phosphorylation of EBP50 is crucial for cell proliferation and transformation **A.** HeLa cells expressing EGFP-EBP50 were synchronized at G1-S border using double thymidine block and released from the block for 0, 3, 6, or 9 h. Phosphorylation of EBP50 was assayed by Western blotting using phospho-T156 antibody. Phosphorylation of histone H3 at S10 was used as the mitotic marker. Molecular marker (*Mr*): kDa. **B.** Cell proliferation assayed using MTS reagent. Data are means ± SEM of 3 independent experiments. **C.** Photos and counting of cresyl violet-stained colonies formed in soft agar assay. **D.** Cells were injected subcutaneously into 8 weeks old NOD/SCID mice. Tumor volumes were measured in series for 4 weeks before the tumor were removed for photographing. Data are means ± SEM. * *p* < 0.05, ** *p* < 0.01, *** *p* < 0.001, n.s. not significant, Student's *t*-test.

As RSK1-mediated phosphorylation of EBP50 at T156 was enhanced in serum- or growth factor-stimulated cells (Figure 3B-3C), we hypothesized that this phosphorylation is a cell cycle-dependent event. Indeed, EBP50 phosphorylation at T156 was not detected in cells that were synchronized at the G1-S border using double thymidine block (Figure 5A). Intriguingly, T156 phosphorylation of EBP50 was evident in cells that were released from thymidine block for 9 h, when cells were at the mitotic phase of the cell cycle as marked by phosphorylation of histone H3 at S10 (Figure 5A). Cell cycle-dependent phosphorylation of EBP50 was also confirmed in mitosis-arrested HeLa cells and SW480 cells (Figure S5A-B). The EBP50 phosphorylation during mitosis was abolished by RSK inhibitor BI-D1870 (Figure S5C). Furthermore, RSK1 activity itself was apparently enhanced in mitotic cells as indicated by its phosphorylation at S380 and phosphorylation of its substrate, the ribosomal protein S6 (rpS6) (Figure S5C-D). These results suggest that EBP50 phosphorylation in mitotic cells is RSK1-dependent. Moreover, PKC-mediated phosphorylation of EBP50 at S339-340, which regulates the affinity between the PDZ domains of EBP50 and the PDZ ligands and thus modulates mobilization of membrane-bound EBP50 to cytosolic compartment [9, 36, 37] was also enhanced in mitotic cells (Figure S5A-B). These results prompted us to explore the biological role of EBP50 phosphorylation during cell proliferation and growth. The results from cell proliferation assays, either using the calorimetric method (Figure 5B) or the dye exclusion method (Figure S5E) indicated a crucial role of EBP50 phosphorylation at T156 in HeLa cells proliferation. The ability to grow in the absence of anchorage to the extracellular matrix is a hallmark of transformed cells [38]. Accordingly, HeLa cells expressing T156A mutant formed fewer colonies in soft agar assay compared to cells expressing wild type EBP50 (Figure 5C). Furthermore, forced nuclear expression of EBP50 by tetracycline regulatory expression markedly increased the anchorage-independent growth potential on differentiated Madin-Darby canine kidney (MDCK) cells (Figure S5F). These *in vitro* assays suggest an essential role of EBP50 for cellular transformation. To further confirm this finding *in vivo*, we performed tumor xenograft study, in which mice injected with cells expressing the phospho-resistant T156A mutant developed smaller tumors (Figure 5D) indicating that T156 phosphorylation is a critical event for the tumor-promoting effect of EBP50.

## DISCUSSION

**Figure 6:**
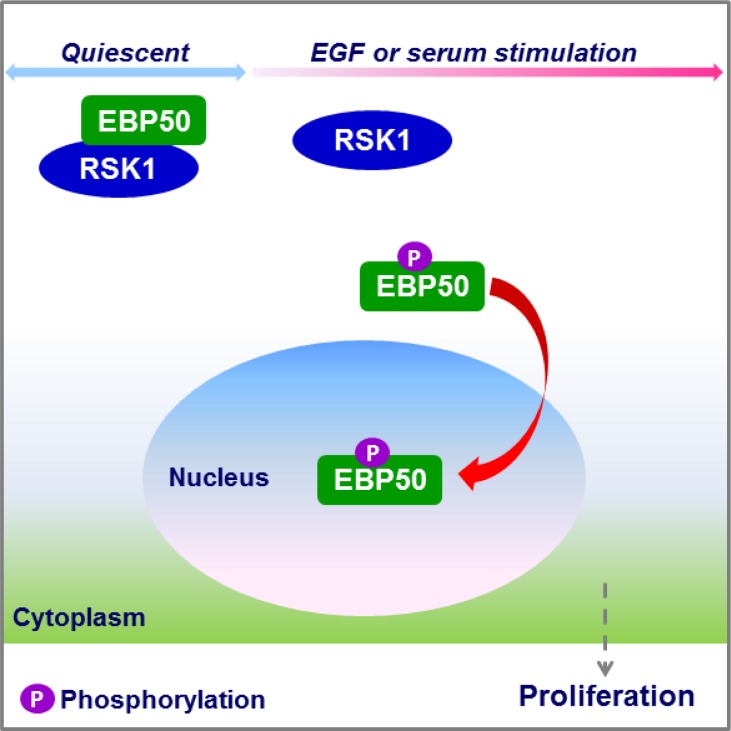
Schematic diagram showing the role of RSK1 in nuclear accumulation of EBP50 RSK1 binds to EBP50 in resting cells, but mitogen-activated RSK1 phosphorylates EBP50, an event which enhances nuclear translocation of EBP50 to facilitate cell proliferation.

The prominent role of RSK1 in nuclear localization of EBP50 was, for the first time, uncovered in this study by using both RNAi and biochemical approaches. RSK1 binds to EBP50 at its first PDZ domain, and mitogen-activated RSK1 phosphorylates EBP50 at T156, an event that is crucial for its nuclear localization (Figure 6).

EBP50 is a highly phosphorylated protein modified by PKC [9, 36], Cdc2 [39], and GRK6A kinases [29]. Phosphorylation of EBP50 has been suggested to impact its scaffolding function, through alteration of its oligomeric state and binding activity [36, 37, 40]. However, the integrative action of these kinases in phosphorylation of EBP50, as well as the subcellular distribution and specific cellular function of distinct phospho-forms of EBP50 remain elusive. Our results indicate that phosphorylation of EBP50 at T156 occurs during mitosis (Figures 5A and S5A-D). Moreover, phosphorylation of EBP50 at S339-340, the sites of PKC action, also happens during mitosis (Figure S5A-B). EBP50 phosphorylation by Cdc2 at S279 and S301 is another mitosis-phase dependent event, and affects its association with Pin1, a peptidyl-prolyl isomerase that its overexpression leads to G2-phase arrest of yeast and HeLa cells [41]. Cdc2-mediated phosphorylation of EBP50 was shown to regulate actin cytoskeleton reorganization and plays an essential role in cytokinesis [39]. In interphase BPAEC, a phospho-mimetic mutant of EBP50 at the Cdc2 phosphorylation sites was distributed in the cytoplasm while wild type protein was predominantly in the nucleus [42]. In contrast, we observed increased nuclear localization of T156E mutant in HeLa cells. These findings suggest that EBP50, existing as distinct phospho-forms that are localized in different subcellular compartments, could be a signaling nexus which finely integrates those kinases and phosphatases critically involved in the mitosis progression.

Correlation of nuclear EBP50 with tumorigenesis was documented for hepatocellular carcinoma and colorectal carcinoma [14, 15]. Previously, we reported that nuclear EBP50 promotes colorectal carcinogenesis by modulating the interaction between β-catenin and TCF1 for activation of Wnt signaling pathway [15]. In the present study, we demonstrate that oncogenic RSK1, which act through its kinase activity, is the signal specific for nuclear accumulation of EBP50 in HeLa cells. The Wnt-β-catenin and Ras-ERK-RSK pathways, though acting in response to different stimuli *via* distinct receptors and effectors, are both important proliferative signals in tumorigenesis. The interactions between these two oncogenic signaling pathways were reported in intestinal [43, 44], prostate [45, 46], lung [47] and pancreatic tumorigenesis [48]. A study in HEK293 cells was shown that the negative regulator of Wnt-β-catenin signaling pathway, glycogen synthase 3β, phosphorylates H-Ras and contributes to its degradation [43]. Furthermore, Ras stabilization by aberrant activation of Wnt-β-catenin signaling was shown related to intestinal tumorigenesis [43]. Our study sheds light on an addition cross-talk between Ras-RSK1 and Wnt-β-catenin signaling which is linked by nuclear entry of EBP50.

Increased nuclear EBP50 was observed in HeLa cells treated with LMB (Figure S4C), an inhibitor of proteins nuclear export that blocks the binding of CRM1 to proteins containing the nuclear export signal (NES), through the interaction with cysteine residue of CRM1 control conserved region [35]. CRM1, a member of the family of importin β-related nuclear transport receptor, has been reported to export certain type of RNA [49, 50], and proteins that bear the leucine-rich NES [51] from the nucleus. However, sequence analysis using NESbase 1.0 search program [52] showed that EBP50 does not contain the classical NES for interaction with CRM1, a prerequisite for CRM1 cargo proteins. This implies that EBP50 could possibly be exported from the nucleus through a cryptic binding with CRM1 or alternatively through interacting partners of EBP50, which contain the NES such as β-catenin [53], EGFR [54] and GRK6A [55].

Dual function proteins, like EBP50, may execute essential physiological functions at one particular cellular compartment, while also play unwanted pathological roles when they wander to another subcellular localization. Thus, a therapeutic approach targeting directly at diminishing such versatile proteins might compromise their normal physiological function; however, prevention of their mislocalization would be an ideal option to preserve essential cellular functions. Our finding not only deciphers the molecular mechanism for nuclear localization of EBP50, a mislocalization that contributes to its pro-proliferative role, but also identifies RSK1 as a potential target to prevent aberrant subcellular trafficking of EBP50, a phenomenon commonly occurring in advanced malignancies.

## MATERIALS AND METHODS

### Plasmids

The EBP50 cDNA was cloned into pEGFP-C1 vector (BD Biosciences, Lexington, KY, USA) at *Xho*I and *BamH*I sites to generate a plasmid expressing EGFP-tagged EBP50 (pEGFP-EBP50). Mutations in the phosphorylation motif of EBP50 were introduced using mutagenic primers and PCR using pEGFP-EBP50 as a template (Table S2). Plasmids expressing full-length EBP50 and its individual PDZ domains were constructed by in-frame cloning of the corresponding cDNA fragments into pGEX vector (Clontech, Mountain View, CA, USA). All the above constructs were sequence verified. Plasmids expressing HA-tagged wild type RSK1-3, and myristoylated constitutively active (Myr-RSK1) and kinase dead (RSK1-KD) mutants of RSK1 were kind gifts from Philippe Roux (University of Montreal, Montreal, Canada). Plasmid expressing HA-tagged constitutively active Ras (HA-RasV12) was kind gift from Hong-Chen Chen (National Chung Hsing University, Taichung, Taiwan). Plasmids expressing Flag-tagged mouse RSK1 was kind gift from Yasuo Watanabe (Showa Pharmaceutical University, Tokyo, Japan).

### Antibodies and chemicals

The following antibodies were used: mouse anti-EBP50 antibody (BD Biosciences, San Jose, CA, USA), rabbit anti-RSK1 antibody, rabbit anti-phospho RXRXXS/T antibody, rabbit anti-rpS6 antibody and rabbit anti-phospho rpS6 (S235-236) antibody (Cell Signaling Technology, Beverly, MA, USA), rabbit anti-phospho RSK1 (S380) antibody (Millipore, Billerica, MA, USA), mouse anti-HA antibody (Covance, Princeton, NJ, USA), mouse anti-α-tubulin antibody (gift from Sheng-Chung Lee, National Taiwan University, Taipei, Taiwan), mouse anti-Ezrin antibody (Lab Vision, Fremont, CA, USA), rabbit anti-GFP antibody and mouse anti-GFP antibody (GeneTex, Hsinchu, Taiwan), mouse anti-Myc antibody (9E10 hybridoma clone from ATCC, Manassas, VA, USA). Anti-phospho-EBP50 (T156) antibody was generated by immunizing rabbits with a synthetic phosphopeptide (LRPRLS(pT)MKKGPSC) and was purified using affinity chromatography. Rabbit anti-EBP50 antibodies (K31 and O40) were generated by immunizing rabbits with a synthetic peptide (MARERAHQKRSSKRC or LQKLGVPVREELLRAQC) and were purified using affinity chromatography. The following chemicals were used: protease inhibitor cocktail, thymidine, nocodazole and Leptomycin B (Sigma-Aldrich, St. Louis, MO, USA), phosphatase inhibitor cocktail (Roche Diagnostic, Basel, Switzerland), purified human EGF (Invitrogen), and BI-D1870 (gift from MedChem Express, Princeton, NJ, USA).

### Cell culture and transfection

HeLa, HEK293, and SW480 cells were cultured in Dulbecco's modified Eagle's medium (DMEM) supplemented with 10% fetal bovine serum and penicillin/streptomycin/ampicillin at 37°C in a humidified incubator containing 5% CO_2_. Plasmids were delivered to the cells using Lipofectamine 2000 (Invitrogen, Carlsbad, CA, USA) according to the manufacturer's instructions. HeLa cells stably expressing EGFP-EBP50 (HeLa-EGFP-EBP50 cells) were selected with 500 μg/ml of G418 (Invitrogen). To generate HeLa cells that stably expressed the phospho-mutants of EBP50 at T156, cells were first infected with EBP50 shRNA lentivirus (Table S2) to knockdown endogenous EBP50 and selected with 1 μg/ml of puromycin. After the knockdown efficiency of EBP50 was confirmed, these stable cells were transfected with shRNA resistant phospho-mutants and wild type EBP50 expressing plasmids following G418 selection. The protein expression levels of these cells were analyzed by Western blotting (Figure S4B).

### RNAi screening

A human shRNA library encoding enzymes involved in phosphorylation, ubiquitination and palmitoylation were obtained from the Academia Sinica RNAi Core Facility, Taipei, Taiwan (Table S1). In this library, individual genes are targeted by 3 to 5 independent short hairpin sequences carried in lentiviral vectors that are arrayed in 96-well plates. HeLa-EGFP-EBP50 cells were plated a day before lentiviruses infection using Biomek FX-P Laboratory Automation Workstation (Beckman Coulter, Brea, CA, USA). At 24 h post-infection, the transduced cells were selected with puromycin for 72 h, and then fixed with 3.7% (w/v) paraformaldehyde (PFA) before being imaged using Cellomics Arrayscan VTI High Content System (Thermo Scientific, Waltham, MA, USA). The equipped software (Molecular Translocation Bioapplication) was used for image analysis. The mean difference in average EGFP fluorescence intensity between the nucleus and the cytoplasm (EGFP N-C) of the triplicate experiment was used to quantify the nuclear translocation of EBP50. The well-to-well variation of RNAi phenotypes in this 96-well format screen was analyzed in HeLa-EGFP-EBP50 cells that infected with lentivirus encoding an empty vector, shVC (Figure S1B).

### Immunofluorescence staining and microscopy

HeLa cells plated onto coverslips the night before the experiments were washed twice with phosphate-buffered saline (PBS) and then fixed with 3.7% (w/v) PFA. Permeabilization was carried out by incubation with CSK buffer (50 mM NaCl, 300 mM sucrose, 10 mM Pipes at pH 6.8, 3 mM MgCl_2_, and 0.5% (v/v) Triton-X100) at room temperature (RT) for 10 min. Cells were then washed twice with PBS and blocked with PBS containing 1% (w/v) BSA, 10% (v/v) goat serum and 50 mM NH_4_Cl at RT for 1 h. After washing briefly with PBS containing 0.2% (w/v) BSA, the cells were incubated with primary antibody at RT for 1 h. Cells were then washed thrice with PBS containing 0.2% (w/v) BSA, and incubated with secondary antibody at RT for 1 h. After washing thrice with PBS containing 0.2% (w/v) BSA, cells were mounted in Vectashield medium (Vector Laboratories, Burlingame, CA). Images were acquired with Zeiss LSM780 confocal microscope (Carl Zeiss Jena GmbH, Jena, Germany) using a 40x or 63x oil immersion objective.

### Immunoprecipitation and Western blotting

Cells were lysed in CSK buffer containing protease and phosphatase inhibitor cocktails plus 1 mM DTT at 4°C for 1 h. Clarified cell lysates containing equal amounts of protein were incubated with protein A bead-bound antibody at 4°C for 4 h. Beads were then washed for three times with CSK buffer and boiled in 2x SDS sample buffer. The immunoprecipitated materials were separated by SDS-PAGE followed by Western blotting analysis using appropriate antibodies. For Western blotting, proteins were blotted onto nitrocellulose membrane and blocking was performed in 5% milk/PBST (PBS containing 0.05% Tween-20). The membranes were incubated with primary antibody overnight at 4°C followed by washes thrice with PBST and vigorous shaking. Then, the membranes were incubated with horseradish peroxidase-conjugated secondary antibodies (GE Healthcare, Buckinghamshire, UK) at RT for 1 h, and visualization was performed using enhanced chemiluminescence reagents (Perkin Elmer, Waltham, MA, USA) and exposure to X-ray film (GE Healthcare). When using phosphospecific antibodies as the primary antibody to detect phosphorylated antigens, the buffer system was replaced with Tris-buffered saline (TBS).

### GST pull-down assay

The recombinant proteins of GST-fused full-length EBP50 and its individual PDZ domains were produced in *Escherichia coli* BL21 strain and conventionally purified on glutathione-Sepharose 4B beads (GE Healthcare) in PBS containing 4 mM 2-Mercaptoethanol (Sigma-Aldrich), 1% TritonX-100 and protease inhibitor cocktail. For interaction between EBP50 and RSKs, HeLa or HEK293 cells that were transfected with plasmids expressing RSKs were lysed in CSK buffer. GST, GST-EBP50, GST-PDZ1 or GST-PDZ2 captured on Sepharose 4B beads was mixed with the respective cell lysate and incubated overnight at 4°C. The beads were then washed with CSK buffer three times with intermittent sedimentation by centrifugation. Pulled down proteins collected by boiling in SDS sample buffer were resolved by SDS-PAGE and analyzed by Western blotting.

### *In vitro* kinase assay

HA-tagged RSKs were immunoprecipitated from transfected HEK293 cells that were lysed in CSK buffer. Immunoprecipitates were then washed twice in CSK buffer and twice in kinase buffer (25 mM Tris at pH 7.5, 2 mM DTT, 10 mM MgCl_2_, and phosphatase inhibitor cocktail). Kinase assays were carried out with purified GST-EBP50 at 37°C for 30 min in the kinase buffer supplemented with 0.5 mM ATP. The samples were subjected to SDS-PAGE and Western blotting analysis.

### FRAP experiment

For nuclear import analysis, nuclear export was blocked in serum-starved cells with 10 ng/ml of LMB 1 h before experiments. FRAP assays were carried out on Zeiss LSM780 confocal microscope with an environmental chamber maintained at 37°C and 5% CO_2_. Bleaching was performed at 100% laser intensity on the entire nuclear fluorescence using 30 cycles. The recovery of fluorescence was recorded by taking images at 10 s interval using a 63x oil immersion objective. Mean nuclear fluorescence intensities were measured using the region of interest (ROI) function of ZEN 2011 software (Carl Zeiss Jena GmbH) after background subtraction. Nuclear fluorescence recovery was calculated by setting the mean nuclear fluorescence of bleached image as 1. The analysis was performed on 14 cells for each stable clone.

### Cell synchronization

HeLa or SW480 cells were synchronized at G1/S phase and mitotic phase using double thymidine block and thymidine-nocodazole block, respectively. For double thymidine block, cells plated at approximately 25% confluency were incubated with 2 mM thymidine for 18 h, released from the first thymidine block for 9 h by washing twice with PBS, and lastly incubated with 2 mM thymidine for 15 h to block the cells at G1/S phase. For the thymidine-nocodazole block, cells were plated at approximately 40% confluency, incubated with 2 mM thymidine for 24 h, and released from the thymidine block for 3 h before incubated with 100 ng/ml nocodazole for 12 h.

### Cell proliferation assay

Cell proliferation was assayed using commercially available MTS tetrazolium reagent, the CellTiter 96^®^ Aqueous One Solution (Promega, Madison, WI, USA). Briefly, 1 × 10^3^ cells were seeded in 96-well plates and incubated for the indicated periods before 20 μl MTS reagent were added. The absorbance at 490 nm was recorded after 4 h incubation with MTS reagent. The experiment was carried out in triplicates.

### Soft agar assay

1.5 × 10^3^ cells were mixed with DMEM containing 0.4% agar (Lonza, Basel, Switzerland) and 10% FBS. The cells suspension was then poured onto a solidified 0.6% agar that prepared in 24-well plates. Cells were incubated for 12 days before stained with 0.05% cresyl violet (Sigma-Aldrich) for colonies counting.

### *In vivo* tumorigenesis study

Cells were trypsinized, washed with PBS and suspended in DMEM/Matrigel basement membrane matrix (BD Biosciences) at a ratio of 1:1 (v/v). 5 × 10^6^ cells were then injected subcutaneously at the dorsal sites of 8 weeks old NOD/SCID mice. The animals were monitored for tumor growth, and tumor sizes were measured 4 weeks after injection. All animals were treated according to guidelines for the experimental animal use specified by the National Taiwan University College of Medicine.

### Data analysis

Data analysis was performed using Excel 2010. All data are presented as mean ± SEM. Differences in the mean values between 2 groups were determined by Student's *t*-test, and *p* value less than 0.05 is considered statistically significant.

## SUPPLEMENTARY MATERIAL FIGURES AND TABLES






