# Supplementary material for: Ras-activated RSK1 phosphorylates EBP50 to regulate its nuclear localization and promote cell proliferation
Source: Oncotarget. 2016 Feb 3;7(9):10283–96. doi: 10.18632/oncotarget.7184 (PMC4891120; doi:10.18632/oncotarget.7184)
Supplement: Supplementary file 1 [file oncotarget-07-10283-s001.pdf]

Ras-activated RSK1 phosphorylates EBP50 to regulate its nuclear localization and promote cell proliferation

Supplementary Material

A

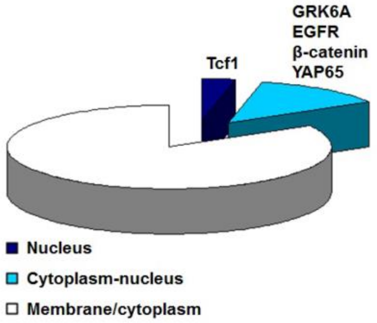

B

Well-to-well Variation	Mean (N-C)	SD	CV (%)
shVC-plate 1	167.0979	8.104473	4.85
shVC-plate 2	161.2840	7.147492	4.43
shVC-plate 3	163.8348	8.785742	5.36

C

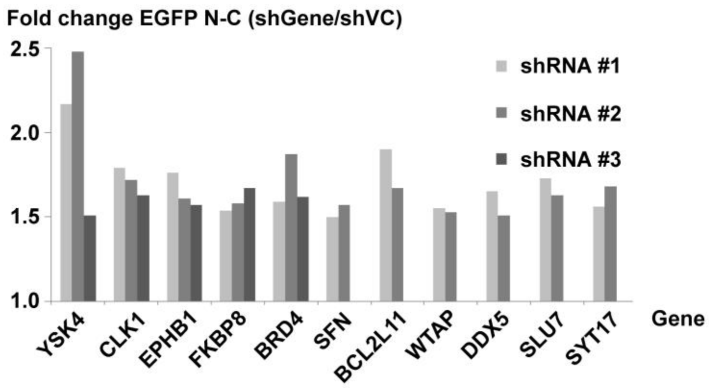

D

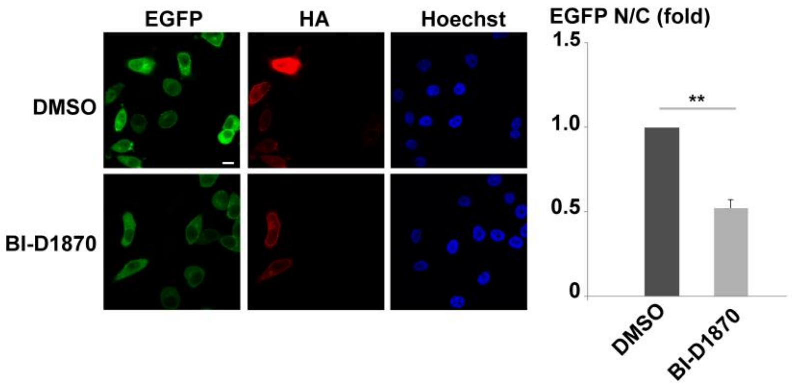

Figure S1: A RNAi screening aimed for genes regulating the nucleocytoplasmic shuttling of EBP50. (**A**) Literature-based classification of interacting partners of EBP50 according to their subcellular localization. (**B**) HeLa-EGFP-EBP50 cells that plated in 96-well plates were infected with control lentivirus (shVC), and the mean of N-C difference of the EGFP signal across a plate

was measured as described in the methodology section. The standard deviation (SD) and the coefficient of variation (CV) were calculated with Excel 2010. (**C**) The candidate genes in which its depletion enhanced nuclear accumulation of EBP50. (**D**) HeLa-EGFP-EBP50 cells transiently expressing HA-RasV12 were treated with dimethyl sulfoxide (DMSO) or BI-D1870 (10 µM) for 30 min, stained with anti-HA antibody, and N/C ratio of the EGFP signal was measured within the cells expressing HA-RasV12. Hoechst 33342 was used to label nuclear DNA. Scale bar: 10 µm. Data are means ± SEM of > 30 cells. ** $p < 0.01$, Student's *t*-test.

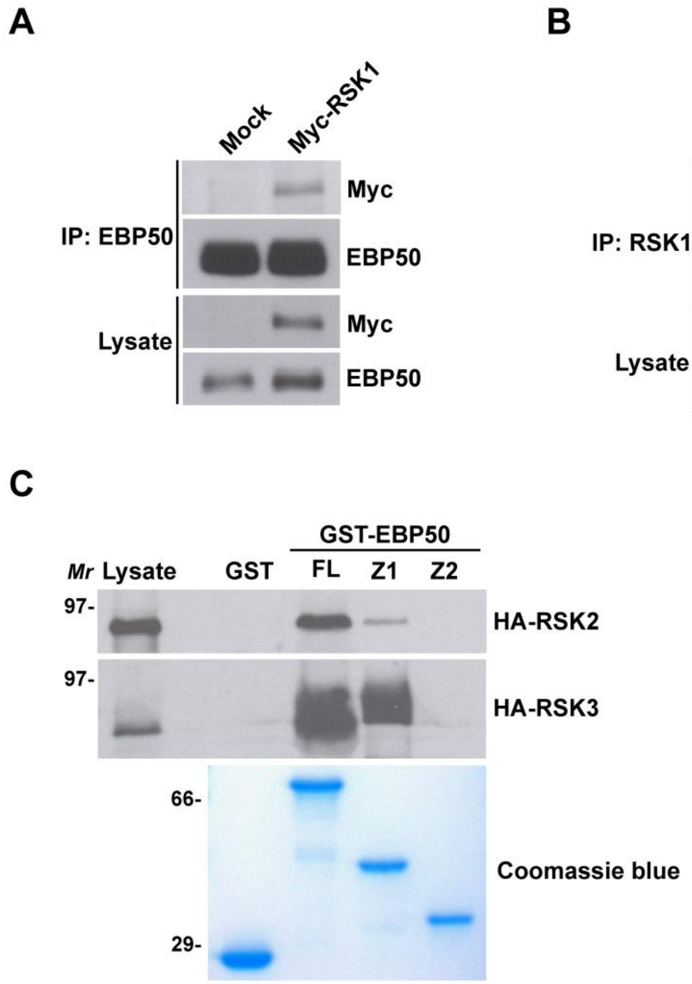

Figure S2: EBP50 interacted with the RSK family of kinases. (**A**) The cellular lysates from HeLa cells that were transfected with Myc-RSK1 or the empty vector were immunoprecipitated with EBP50 antibody and the co-immunoprecipitated RSK1 was analyzed by Western blotting using anti-Myc antibody. (**B**) HeLa cells were transfected with EGFP-EBP50 or an empty vector. Then, endogenous RSK1 was immunoprecipitated from the cell lysates and the immunoprecipitated materials were analyzed by Western blotting using anti-EBP50 antibody. (**C**) Cell lysates from HEK293 cells transiently expressing HA-tagged RSK2 or RSK3 were subjected for *in vitro* pull-down assay with GST-fused full-length (FL), first PDZ domain (Z1) or second PDZ domain (Z2) of EBP50. The pulled down materials were separated by SDS-PAGE and analyzed by Western blotting using anti-HA antibody. The coomassie blue-stained gel showed the expression and comparable loading of each recombinant protein used in the assay. Molecular marker (*Mr*): kDa.

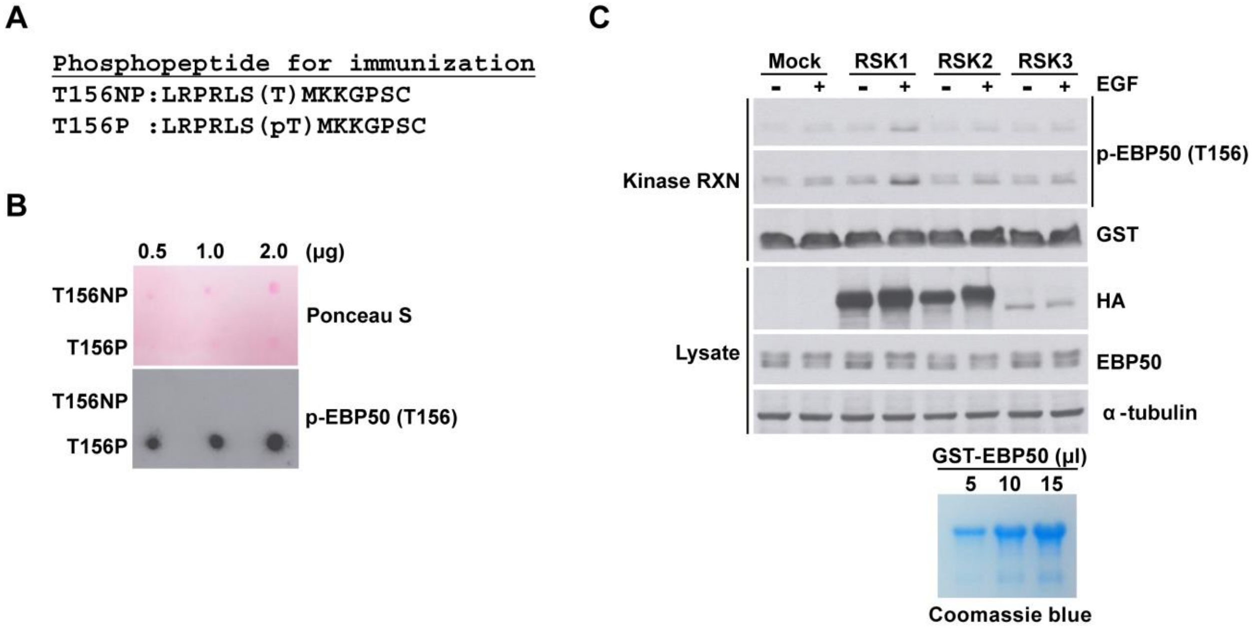

Figure S3: Generation and analysis of anti-phospho-EBP50 (T156) antibody. (**A**) The amino acids sequence of synthetic phosphopeptide (T156P) used to generate site and phosphorylation state-specific antibody for EBP50. A control peptide (T156NP) was also synthesized in which T156 was not phosphorylated. (**B**) Dot blot analysis to assess the production of a phospho-specific antibody, p-EBP50 (T156), for application in studies on phosphorylation of EBP50 at T156. (**C**) *In vitro* kinase assays were performed similarly to what was described in Figure 3D. In brief, HEK293 cells were transfected with HA-tagged RSK1, RSK2, RSK3, or an empty vector, serum starved, and stimulated with EGF (50 ng/ml) for 15 min. The immunoprecipitated RSKs was incubated with 5 μl purified GST-EBP50, as shown by the coomassie blue stained gel, and phosphorylation of EBP50 after 30 min of kinase reaction at 37°C was analyzed by immunoblotting using phospho-T156 antibody, p-EBP50 (T156), as shown by two images that were obtained at different film exposure times.

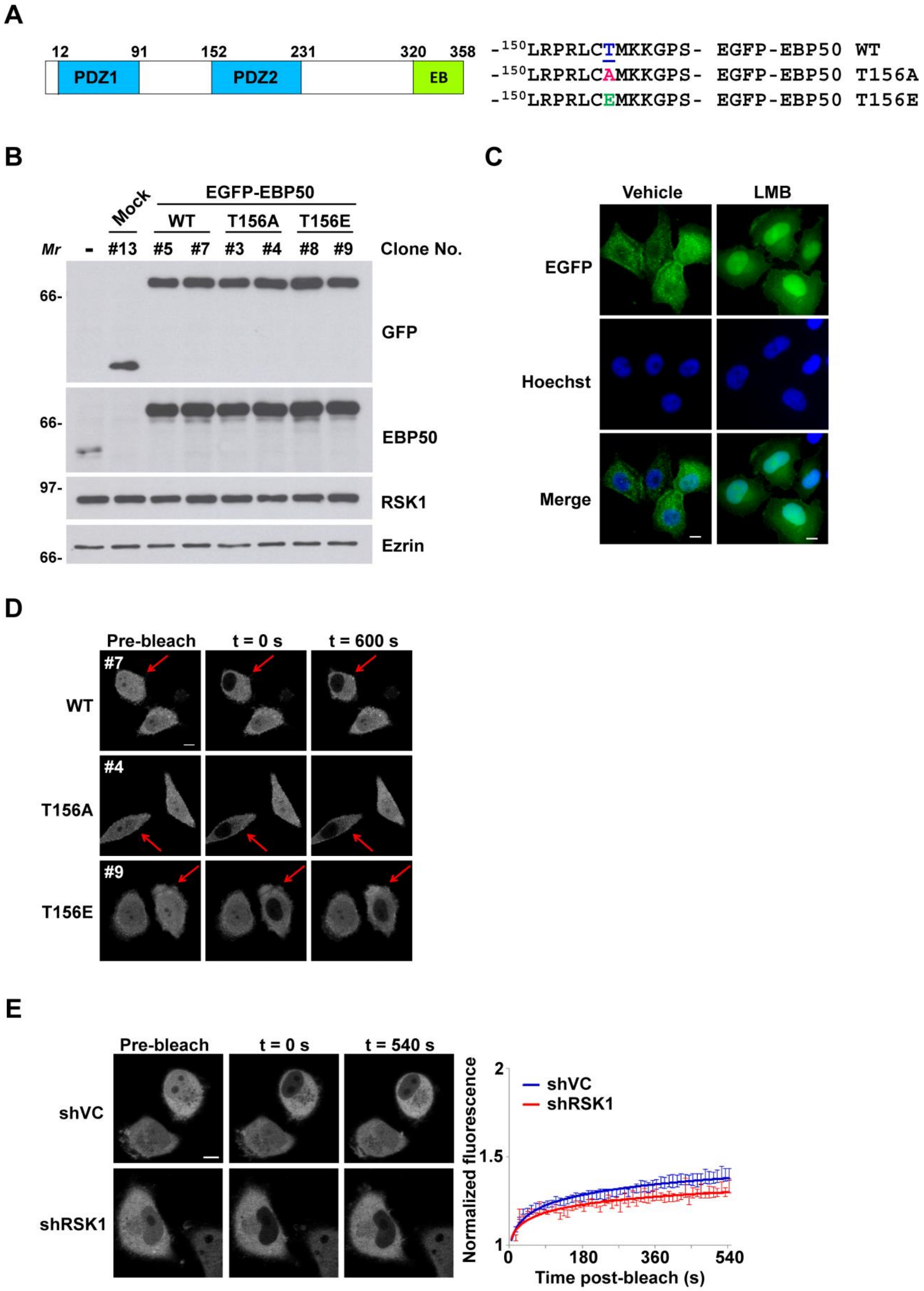

Figure S4: Phosphorylation of EBP50 at T156 is related to increased nuclear localization.

(**A**) Schematic diagram of phospho-mutants of human EBP50 (WT) in which threonine residue

at position 156 within the RXRXXpS/T motif were replaced with either alanine (T156A) or glutamate (T156E) as indicated. (**B**) Western blotting analysis using both GFP and EBP50 antibodies to detect both exogenous and endogenous protein expression levels in HeLa clones stably re-expressing EGFP-tagged wild type EBP50 and its phospho-mutants at T156. Ezrin and RSK1 were examined as the loading controls. Molecular marker (*Mr*): kDa. (**C**) HeLa cells transiently expressing EGFP-EBP50 was serum-starved before being treated with 10 ng/ml of LMB. Scale bar: 10 µm. (**D**) Photobleaching nuclear import assay on phospho-mutants of EBP50. Fluorescence images of typical cells from each clone at pre- or post-bleach times and at experimental end points. Scale bar: 10 µm. Quantitative data for recovery of fluorescence is shown in Figure 4B. (**E**) Photobleaching nuclear import assays on RSK1-knocked down HeLa cells and mock-transduced cells (shVC) that were co-transfected with HA-RasV12 and EGFP-EBP50. These cells were grown in complete medium with 10% FBS and treated with LMB (10 ng/ml) for an hour before experiments. RasV12-expressing cells were selected based on co-transfection with the EGFP-EBP50 plasmid. Fluorescence images of representative cells from each clone at pre-, post-bleach, and at end points were shown. Quantitative data of the photobleached-cells for recovery of nuclear fluorescence are means $\pm$ SEM of 10 cells for each time points. Scale bar: 10 µm.

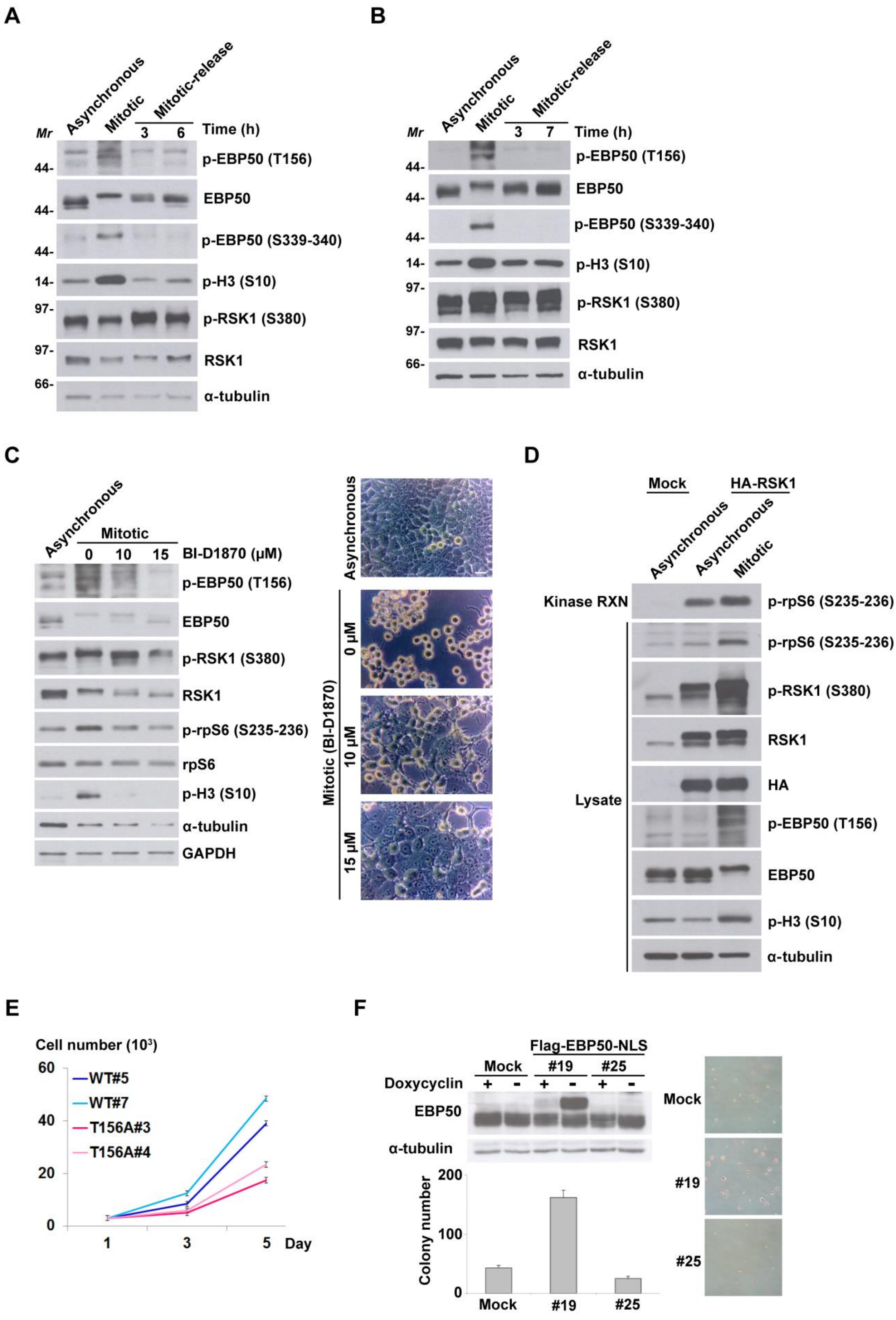
A
Asynchronous
Mitotic
Mitotic-release
Mr
3
6
Time (h)
p-EBP50 (T156)
EBP50
p-EBP50 (S339-340)
p-H3 (S10)
p-RSK1 (S380)
RSK1
α-tubulin
44-
14-
97-
66-
B
Asynchronous
Mitotic
Mitotic-release
Mr
3
7
Time (h)
p-EBP50 (T156)
EBP50
p-EBP50 (S339-340)
p-H3 (S10)
p-RSK1 (S380)
RSK1
α-tubulin
C
Asynchronous
Mitotic
0
10
15
BI-D1870 (μM)
p-EBP50 (T156)
EBP50
p-RSK1 (S380)
RSK1
p-rpS6 (S235-236)
rpS6
p-H3 (S10)
α-tubulin
GAPDH
Mitotic (BI-D1870)
0 μM
10 μM
15 μM
D
Mock
HA-RSK1
Asynchronous
Asynchronous
Mitotic
Kinase RXN
Lysate
p-rpS6 (S235-236)
p-rpS6 (S235-236)
p-RSK1 (S380)
RSK1
HA
p-EBP50 (T156)
EBP50
p-H3 (S10)
α-tubulin
E
Cell number (10³)
60
40
20
0
WT#5
WT#7
T156A#3
T156A#4
1
3
5
Day
F
Flag-EBP50-NLS
Mock
#19
#25
Doxycyclin
+
-
+
-
+
-
EBP50
α-tubulin
Colony number
200
100
0
Mock
#19
#25
Mock
#19
#25

Figure S5: Cell cycle-dependent phosphorylation of EBP50 is crucial for cell proliferation and transformation. HeLa cells (**A**) or SW480 cells (**B**) were synchronized at mitotic phase using thymidine-nocodazole block (mitotic) and released from the block (mitotic release) for 6h (**A**) or 7h (**B**). Phosphorylation of EBP50 was assayed by Western blotting using phospho-T156 and phospho-S339-340 antibodies. Phosphorylation of histone H3 at S10 was used as the mitotic marker. Molecular marker (*Mr*): kDa. (**C**) HeLa cells synchronized at mitotic phase were treated with BI-D1870 at indicated concentrations. Phosphorylation of EBP50 was assayed by Western blotting using p-EBP50 (T156) antibody. Phosphorylation of RSK1 at S380 and ribosomal protein S6 (rpS6) at S235-236 were assayed to examine activation of RSK1 at mitotic phase. GAPDH or α-tubulin was examined as a loading control. The morphology of cells at the moment of harvest for the above experiment is shown in photographs. Scale bar: 20 µm. (**D**) HeLa cells transiently expressing HA-RSK1 were synchronized at mitotic phase or left asynchronous. RSK1 was immunoprecipitated from these cells and processed for *in vitro* kinase assay using synthetic peptide of rpS6 (Millipore). Phosphorylation at S235-236 of both synthetic peptide after kinase reaction and endogenous rpS6 in cellular lysates before immunoprecipitation were assessed by Western blotting. (**E**) Trypan blue exclusion analysis to examine the growth of HeLa cells expressing wild type and T156A mutant of EBP50; each with two independent stable clones. (**F**) Soft agar assay for MDCK cells stably expressing amino terminally Flag-tagged and carboxyl terminally NLS-fused EBP50 under the tetracycline-repressible system. Western blotting analysis demonstrated one stable clone (#19) expressed a comparable level of NLS-fused EBP50 to endogenous EBP50 when doxycycline was removed. Another stable clone (#25) which failed to express the exogenous protein and a mock transfectant were used as negative controls when all cell lines were cultured in the absence of doxycycline for soft agar assays (photographs and histograms).
