# Supplementary material for: Ras-activated RSK1 phosphorylates EBP50 to regulate its nuclear localization and promote cell proliferation
Source: Oncotarget. 2016 Feb 3;7(9):10283–96. doi: 10.18632/oncotarget.7184 (PMC4891120; doi:10.18632/oncotarget.7184)
Supplement: Supplementary file 3 [file oncotarget-07-10283-s003.pdf]

Table S2. The sequence of primers used to generate plasmid constructs used in this study and shRNA targeted sequence for EBP50- or RSK1-knocked down in HeLa cells

Plasmid	Primer ID	Sequence
pEGFP-EBP50	BMRC100	TATGTTCTCGAGAAATGAGCGCGGACGCAGCGG
	BMRC142	GGATCCTCAGAGGTTGCTGAAGAGTTCGT
pEGFP-EBP50T156A	BMRC100	TATGTTCTCGAGAAATGAGCGCGGACGCAGCGG
	BMRC142	GGATCCTCAGAGGTTGCTGAAGAGTTCGT
	BMRC179	GGCTCTGTGCCATGAAGAAGGGCCC
	BMRC148	TCTTCATGGCACAGAGCCGAGGCCG
pEGFP-EBP50T156E	BMRC100	TATGTTCTCGAGAAATGAGCGCGGACGCAGCGG
	BMRC142	GGATCCTCAGAGGTTGCTGAAGAGTTCGT
	BMRC181	GGCTCTGTGAGATGAAGAAGGGCCC
	BMRC150	TCTTCATCTCACAGAGCCGAGGCCG
shRNA resistant EBP50	BMRC100	TATGTTCTCGAGAAATGAGCGCGGACGCAGCGG
	BMRC142	GGATCCTCAGAGGTTGCTGAAGAGTTCGT
	BMRC183	TCGCGAGACCGATGAATTTTTTAAGAAATGCAGAGTGATC
	BMRC152	AAAAAATTCATCGGTCTCGCGATCCACCACCAGCAGCTTG
shRNA resistant T156A	BMRC100	TATGTTCTCGAGAAATGAGCGCGGACGCAGCGG
	BMRC142	GGATCCTCAGAGGTTGCTGAAGAGTTCGT
	BMRC179	GGCTCTGTGCCATGAAGAAGGGCCC
	BMRC148	TCTTCATGGCACAGAGCCGAGGCCG
	BMRC183	TCGCGAGACCGATGAATTTTTTAAGAAATGCAGAGTGATC
	BMRC152	AAAAAATTCATCGGTCTCGCGATCCACCACCAGCAGCTTG
shRNA resistant T156E	BMRC100	TATGTTCTCGAGAAATGAGCGCGGACGCAGCGG
	BMRC142	GGATCCTCAGAGGTTGCTGAAGAGTTCGT
	BMRC181	GGCTCTGTGAGATGAAGAAGGGCCC
	BMRC150	TCTTCATCTCACAGAGCCGAGGCCG
	BMRC183	TCGCGAGACCGATGAATTTTTTAAGAAATGCAGAGTGATC
	BMRC152	AAAAAATTCATCGGTCTCGCGATCCACCACCAGCAGCTTG

Gene Symbol	Accession No.	shRNA Target Sequence
SLC9A3R1 (EBP50)	NM_004252	CAGGGAAACTGACGAGTTCTT
RPS6KA1 (RSK1)	NM_002953	GCTCTATCTCATTCTGGACTT (shRSK1#2)
		GACCATGACACTGATTCTGAA (shRSK1#5)
